# Supplementary material for: Population Structure of Nation-Wide Rice in Thailand
Source: Rice (N Y). 2021 Oct 24;14:88. doi: 10.1186/s12284-021-00528-2 (PMC8542525; doi:10.1186/s12284-021-00528-2)
Supplement: Supplementary file 2 — Additional file 2. The supplementary figures. [file 12284_2021_528_MOESM2_ESM.pdf]

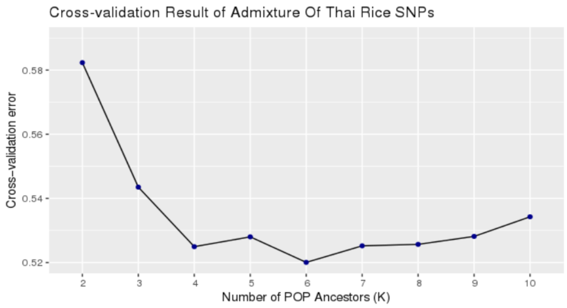

Figure 5 The Elbow method result that was used to find the optimal number of ancestors from ADMIXTURE. The optimal number is 4 ancestors.

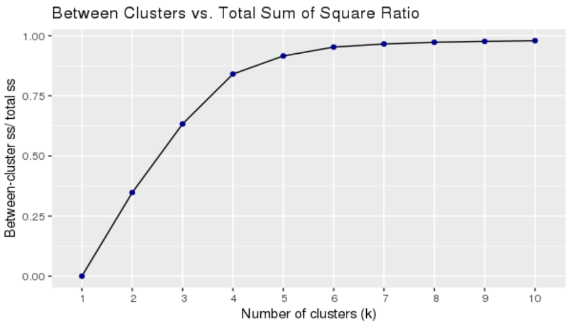

Figure 6 The Elbow method result that was used to find the optimal number of clusters for k-means clustering. The optimal number is 5 clusters.
